# The benefit of a tough skin: bullet holes, weathering and the preservation of heritage

**DOI:** 10.1098/rsos.160335

**Published:** 2017-02-22

**Authors:** Lisa Mol, M. Gomez-Heras, C. Brassey, O. Green, T. Blenkinsop

**Affiliations:** 1 Department of Geography and Environmental Management, University of West England, Bristol, UK; 2 Oxford Rock Breakdown Laboratory, School of Geography and the Environment, University of Oxford, Oxford, UK; 3 Departamento de Geología y Geoquímica, Universidad Autónoma de Madrid, Madrid 28049, Spain; 4 Division of Biology and Conservation Ecology, School of Science and the Environment, Manchester Metropolitan University, Manchester M1 5GD, UK; 5 Department of Earth Sciences, University of Oxford, South Parks Road, Oxford OX1 3AN, UK; 6 School of Earth and Ocean Science, Cardiff University, Cardiff CF10 3AT, UK

**Keywords:** heritage, conservation, materials, weathering

## Abstract

Projectile damage to building stone is a widespread phenomenon. Sites damaged 100 years ago during the First World War still see daily use, while in a more contemporary setting numerous reports show the damage to buildings in Babylon, Mosul and Palmyra. While research has been carried out on the long-term effects of conflict such as fire damage, little is known about the protracted damage sustained through the impact of bullets, shrapnel and other metal projectiles outside of the field of engineering focused on ceramics and metals. To investigate alterations to mineral structure caused by projectile damage, impacts were created in medium-grained, well-compacted, mesoporous sandstone samples using 0.22 calibre lead bullets shot at a distance of 20 m. Half these samples were treated with a surface consolidant (Wacker OH 100), to mimic natural cementation of the rock surface. These samples were then tested for changes to surface hardness and moisture movement during temperature cycles of 15–65°C. Petrographic thin section analysis was carried out to investigate the micro-scale deformation associated with high-speed impact. The results surprisingly show that stress build-up behind pre-existing cementation of the surface, as found in heritage sites that have been exposed to moisture and temperature fluctuations for longer periods of time, can be alleviated with a bullet impact. However, fracture networks and alteration of the mineral matrices still form a weak point within the structure, even at a relatively low impact calibre. This initial study illustrates the need for geomorphologists, geologists, engineers and heritage specialists to work collectively to gain further insights into the long-term impact of higher calibre armed warfare on heritage deterioration.

## 1. Introduction

Damage to immovable heritage frequently occurs during armed conflict and can have a significant detrimental impact on the preservation of these sites. Traces of damage are visible almost everywhere—Tate Britain in London, the Main Building of Cardiff University and the General Post Office in Dublin all bear scars of conflict, to name but a few. News reports of widespread use of weapons in countries such as Iraq [1] and Syria in contemporary conflict have often mentioned the destruction of high-profile heritage such as Palmyra and Mosul, as part of a campaign of deliberate heritage destruction [2,3]. This is a grave cause for concern and while the unrest continues it is difficult to obtain an accurate assessment of the damage incurred. Widespread destruction of heritage as a result of crossfire or deliberate targeting to eradicate the cultural and religious identity of the opposition has sparked new and innovative conservation efforts such as Project Mosul which focuses on three-dimensional digital recreations of destroyed heritage [4].

The preservation of heritage damaged during a conflict represents a specific challenge in conservation, both owing to the extent of the damage, and also because of the ‘meaning’ damage may have for society and the history of a given property; this is illustrated by sites such as the General Post Office in Dublin, where bullet impacts created by the execution of the leaders of the Easter Rising (1916) supersede the building's perceived value ([5], p. 60). Following the process of semiotics, buildings or heritage might evoke different concepts depending on the social constructions society imposes upon them [6]. Because of this social meaning and the symbolic concept of heritage, built heritage is particularly at risk, as it is often targeted during conflicts by those aiming for a ‘symbolic’ destruction of the society that owns the heritage.

**Figure 1.**
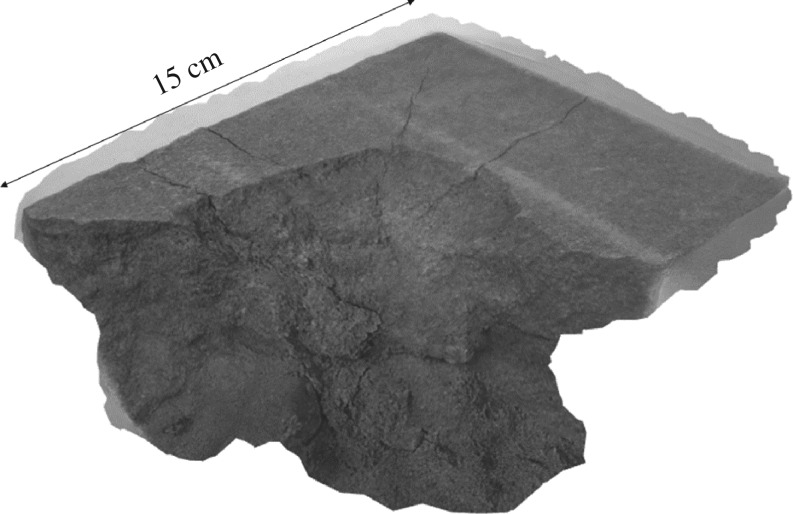
Three-dimensional model of sample block remnant after AK-47 impact.

Furthermore, the nature of warfare has drastically changed over the past century. The introduction of progressively more destructive weaponry such as grenades, bombs and mechanized artillery has increased the risk of damage or destruction of heritage in areas of conflict. Increasing velocity and impact potential of the projectiles used in warfare have resulted in a far greater potential for damage. Stone samples shot with an AK-47 illustrate the destructive effect on one of the test blocks used in this experiment (figure 1), and are a testament to the potential damage to heritage in conflict areas.

While a significant body of engineering research has investigated the impacts of alien objects on surfaces, very little is known about the subsequent deterioration of the material (over decades to centuries). Research into bullet impacts has included targets of composite materials (integral armour; [7,8]) and aluminium [9]. Experiments have also been conducted using granular material such as gravel and crushed stone [10] but there is a noticeable absence of research into impacts on stone surfaces other than theoretical studies [11]. This field of research is predominantly focused on immediate damage assessment, while the biggest concern for damaged stone surfaces in the context of heritage structures is the long-term damage potential through enhanced weathering processes. We do not know, for example, how complex fracture networks created by impacts respond to moisture movement, cementation or to environmental change over the following decades and centuries. Within weathering studies, with the exception of a mention in the Canadian Conservation Institute Agent 1 risk listings of catastrophic forces [12], there is no detailed classification for impact damage, nor does it feature in the literature published by heritage protection organizations ICCROM and UNESCO. The way in which high-speed impacts affect the integrity of the stone matrix is unknown, particularly in relation to the response of any ensuing complex fracture network to environmental change.

Furthermore, the response of a stone surface and subsurface to environmental stressor (such as moisture movement and temperature) changes as surface cementation takes place. Naturally occurring hard, resilient crusts result from the cementation of material by external sources and/or surface accumulation and cementation of endogenous material, potentially drawn to the surface by evaporation [13]. In the context of contemporary conflict, zones within the Middle East are likely to experience considerable temperature fluctuations with high daytime temperatures, resulting in increased capillary forcing of water through the stone surface [14] and increasing the potential for case hardening. The three factors (capillary moisture rise, temperature stress and case hardening) are therefore included as factors in this study.

This investigation explores a number of laboratory approaches to investigate the impact of bullets on stone surfaces and the response of damaged areas to exposure to environmental stressors, as well as fracture propagation and matrix deformation at the microscopic scale. To this end, artificial impacts were created by firing bullets at sandstone samples from a known distance, which were analysed using rock surface hardness, moisture distribution and material loss as indicators of aggravation. To limit variability caused by unknown weathering history it was decided to artificially create case-hardened rock surfaces in freshly quarried blocks rather than obtain samples from existing buildings. Half the samples were therefore treated with a consolidant to provide an analogy for the decreased surface porosity and increased hardness associated with case hardening. All samples were subjected to temperature- and moisture-induced stress to monitor the response of the damaged areas to weathering processes, using case-hardening simulants to investigate the effect of impact after prolonged exposure to environmental processes such as moisture through-flow and mobility and deposition of solutes, as would be expected on a heritage site [15]. Finally, the impacted areas were analysed using microscopy, scanning electron microscopy and micro-computed tomography (μCT) to map micro-scale effects of bullet impacts.

As illustrated in figure 1, the extent of destruction wrought by the AK-47 impact on our sample blocks ensured the remaining fragments were not suitable for a full examination of the interactions between impact sites, surrounding fracture networks and any unharmed material in the periphery. Impacts were therefore scaled down to ensure a full impact was contained within the test blocks, thus permitting high-resolution imaging of the entire sample (see Methodology section). The low-powered rifle used herein undoubtedly underestimates the potential damage associated with contemporary warfare. It does, however, allow for the full impact structure network, its response to environmental stress and the effect of pre-existing hardening of the surface to be mapped within the constraints of sample size. While this discrepancy in scales must be noted, here we lay the methodological foundations for future investigations of larger impacts, and highlight the pressing need for further research into endangered built heritage.

The results presented here highlight that the impact of bullets on stone surfaces is not a straightforward creation of an impact fracture network but is dictated by the stone's ability to process the influx of stress associated with the impact as well as pre-existing alteration of the stone such as case hardening. This knowledge is of particular importance when the object of investigation is a heritage site with a long exposure and weathering history. As increasing numbers of sites are not only caught in crossfire but actively targeted, we urgently need a better interdisciplinary understanding to be able to safeguard heritage for future generations.

## 2. Methodology

### 2.1. Stone samples

Freshly quarried stone, which had experienced no exposure to weathering, was obtained from the Huesca region in northeast Spain. This type of sandstone is well-consolidated mesoporous sandstone (average pore size between 40 and 70 µm; water absorption capacity of 1.8%). Test blocks measured 15 × 15 × 7.5 cm, with the larger 15 × 15 cm surface used as the target. This stone is particularly suitable for this test owing to the homogeneity in matrix distribution, thereby minimizing the potential impact of test anomalies caused by variations in the sandstone.

### 2.2. Pre-impact tests

Before any tests were carried out a Piccolo hardness tester was used to map variability and changes in rock surface hardness. This was done to measure both the impact of the bullet tests but also to account for pre-existing variations within the stone matrix; while the choice of stone for these tests minimizes introduction of natural variability it is possible that the process of quarrying and transportation could have caused weaknesses within the individual stone samples.

The Piccolo hardness tester measures surface hardness as a function of unconfined compressive strength. A 3 mm diameter spherical tungsten carbide test tip is mounted in an impact body and impacts under spring force against the test surface at which it is directed. The velocity before impact (*V*,) and after impact (*V*2) are measured automatically and displayed as a ratio *(V2/V,* ×1000) which is denoted by the unit ‘L’, or Leeb unit (as described for a similar impact device in [16]). Each block was divided into 16 squares measuring 3.75 × 3.75 cm. Within each square four measurements were taken to obtain a total of 64 measurements per block surface. Similar devices have been used widely to monitor loss of rock strength as an expression of weathering process rate [17,18].

**Figure 2.**
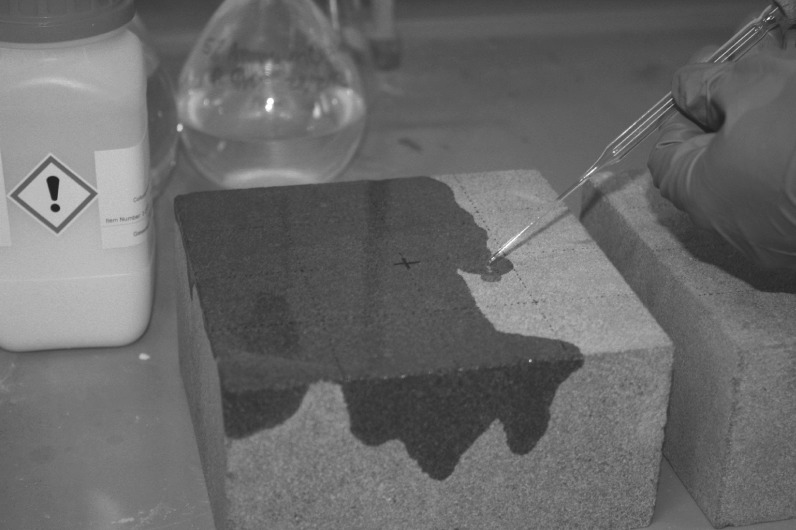
Application of Wacker OH 100 treatment.

**Figure 3.**
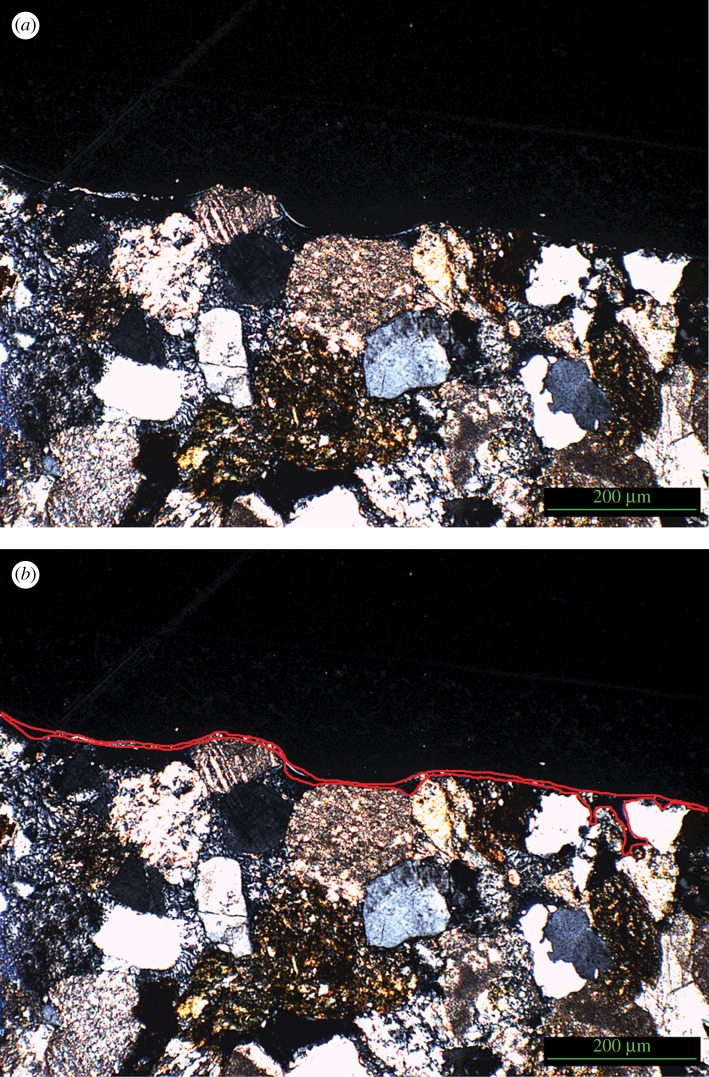
(*a*) Wacker OH 100 gel layer in original image. (*b*) Wacker OH 100 gel layer with accentuated observation of gel deposition.

Cementation of the surface, referred to as ‘case hardening’ and manifested as a surface crust, often develops after freshly cut building stone is exposed to environmental factors such as precipitation and temperature fluctuations [19,20]. This cementation can consist of multiple phases such as carbonate, calcite or iron oxide crusts, and while stabilizing the top surface can also enhance deterioration of the stone matrix owing to subsurface moisture accumulation and subsequent dissolution of the cement matrix as well as increasing pressure on the surface [21–23]. To address this change in surface hardness and porosity, half the samples were treated with Wacker SILRES BS OH 100, an ethyl silicate-based consolidant which forms a silica-based gel upon contact with moisture in the stone pores, and subsequently dries within the pore system [24], by pipetting the substance onto the surface (figure 2). This consolidant is also used in conservation of stone artefacts to increase hydrophobicity and decrease bioinfestation potential [25–27]. One layer was applied to mimic surface consolidation, as it is used here as an analogy for natural case hardening, rather than multiple applications which might have resulted in consolidation of the majority of the sample [28]. After curing, a thin layer of consolidant was observed at the surface of the block but, as anticipated, this layer was mostly restricted to the surface and immediate subsurface (figure 3*a*,*b*). Case hardening under natural circumstances results in the deposition of a hardened surface layer with a reduction in porosity at the region immediately underlying the cemented crust up to a depth of 4 mm [13]. A consolidant which forms a hardened layer at the surface and reduced porosity in the subsurface was, therefore, deemed to be a suitable analogy for natural case hardening, though figure 3 illustrates that in this case the gel layer did not intrude further than 1 mm into the surface. The results of this study, therefore, provide an insight into the potential effect of a thin case-hardened layer on an impacted surface, an effect that could be exacerbated if the layer reaches the thicknesses reported by Viles & Goudie [13].

The samples were left to cure for 5 days before impacts were created on the shooting range. The rock surface hardness was monitored throughout this time to ensure the consolidant had fully hardened and no further increases in rock surface hardness were noted. The rock surface hardness of the sample surfaces was re-measured after impact and after every cycle in the environmental cabinet (see §2.4.1) to quantify relative rates of surface deterioration in all samples.

### 2.3. Projectile impact

**Figure 4.**
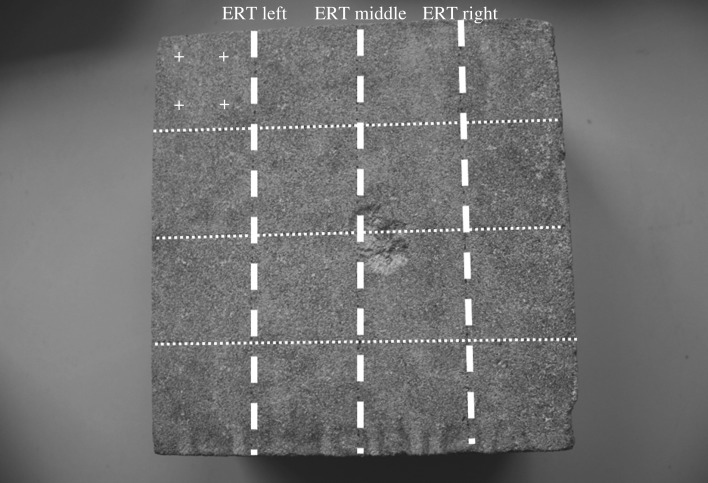
Impact area at sample 6, also showing sampling strategy for the Piccolo and ERT.

Samples 2–4 and 6–8 were taken to the Witney Rifle Club (Oxfordshire, UK). Lead bullets of 0.22 calibre were shot from 20 m distance into the centre of each block, clearly marked during the pre-impact tests, using a standard shoulder-supported rifle. Each centre was hit three times to increase the impact on the stones. In all cases, the impact areas of each shot overlapped, as demonstrated by sample 6 shown in figure 4. This figure also shows the sampling strategy for the Piccolo (as indicated by the crosses, four measurements per square section) and the electric resistivity tomography (ERT) measurements (moisture movement, see post-impact tests).

### 2.4. Post-impact tests

A number of tests were carried out after the impact. Firstly, the samples were re-weighed to determine mass loss during the impact. This was followed up with a three-dimensional scan of impact sites, using a Mephisto Eos 3D scanner with a Canon EOS 1000D camera and Optoma DLP EX 531p projector to obtain visual evidence of the impact site. Once the volume loss was established the samples were subjected to environmental stress to determine response of the damaged area to stressors such as temperature and moisture fluctuations as would be present at an outdoor site where damaged heritage might be situated.

#### 2.4.1. Environmental cabinet tests

To further the investigation into the deterioration of the samples under more extreme environmental conditions the samples were placed in an environmental cabinet (Sony-FE 300H). A programme was designed for six consecutive days with alternating 6 h cycles of 15°C and 65°C which mimic extreme summer temperatures experienced by heritage in arid and semi-arid Middle Eastern environments. Half the samples (3, 4, 7 and 8) were placed in 2 cm of water to mimic groundwater conditions whereas samples 1, 2, 5 and 6 were placed directly within the cabinet without further access to water other than the humidity within the cabinet which was kept low to account for the evaporating water of the immersed samples.

For further clarification of the samples and their treatment, see table 1 for an overview.

**Table 1.** Overview of individual sample treatments.

sample no	Wacker treatment	bullet holes	water immersion in cabinet
1	no	no	no
2	no	yes	no
3	no	yes	yes
4	no	yes	yes
5	yes	no	no
6	yes	yes	no
7	yes	yes	yes
8	yes	yes	yes

The immersion of half the samples in water fulfilled a dual role; firstly to test the hypothesis that a known weakness in the surface would lead to increased evaporation of moisture from that particular area, leading to an increase in deterioration around the area. Previous work by Mol & Viles [14] showed that increased temperatures lead to higher capillary pull through a mass of stone and high surface evaporation. In addition, the internal movement of moisture is known to deteriorate a stone surface [20,29]. This gives a theoretical justification for the assumption that a weakness such as a bullet hole would experience higher deterioration rates under high temperature conditions. However, this has never been tested, nor is the influence of case hardening known in this scenario.

#### 2.4.2. Electric resistivity tomography

It has been established that the environmental stress placed on the samples in the environmental cabinet affected the surface strength of the impact area. Shockey [30] conducted a study on the impact of projectiles on steel and found that underneath the impact area a hemispherical area of deformation was visible. As not many impact tests have been performed on stone surfaces this model will be used, and initially the assumption will be made that underneath the direct impact area a weakened hemispherical area will have developed that could influence moisture movement, as its higher permeability would encourage moisture flow into this area. To test this assumption and monitor movement of moisture a GeoTom (Geolog2000) ERT device was used, which can measure profiles using up to 100 electrodes. Miniaturized cabling attachments with fixed spacing have been developed in the Oxford Rock Breakdown Laboratory which use spring-loaded electrodes permanently spaced at 0.5 cm to measure resistivity at up to 2.5 cm depth, giving a very high-resolution two-dimensional distribution of resistivity. The basic principle of ERT is to pass a 12 V DC current between two designated potential electrodes and measure the transient response. By increasing the spacing between the two designated electrodes the current penetrates the stone further, building up a two-dimensional transect of point- specific resistivity which can then be recalculated into a most probable resistivity distribution using the inversion program RES2DInv [31,32]. Each sample was measured along three vertical transects, the middle one covering the site of impact and the other two transects placed left and right of the centre equidistant between block centre and edge.

#### 2.4.3. Thin section microscopy

To investigate deformation of minerals, petrographic thin sections of the bullet impact sites were produced with added glass coverslips to increase clarity of the images. Imaging was undertaken with a Nikon Optiphot-pol microscope (with transmitted and incident halogen light illumination). The following objectives were used for transmitted light observations: ×4/0.1 160/−; ×10/0.25 160/−; ×40/0.65 160/0.17. M-plan Differential Interference Contrast objectives were used for incident light observations: ×5 0.1 210/0; ×/0.25 210/0; ×40 0.65 210). To produce the images we used a Q-imaging (QICAM fast 1394) high-performance IEEE FireWire 12-bit digital CCD (1392 × 1040, 1.4 million pixels, with a pixel size of 4.65 µm × 4.65 µm) camera system attached by a ½′′ C-mount. Live image capturing and processing was achieved using Syncroscopy's AcQuis (v. 4.0.1.8) software.

#### 2.4.4. X-ray tomography

Sample 2 was cut to encompass only the bullet impact, and to create a subsample of suitable size for µCT at the Manchester X-Ray Imaging Facility (MXIF), University of Manchester. The specimen was scanned in a 320/225 kV Nikon XTEK bay (Nikon Metrology Ltd, Tring, UK) at 300 kV and 50 µA, with 1 s exposure time over 3142 projections. The resulting voxel size was 0.052 µm. The scan was reconstructed using the software CT Pro and imported into Avizo (FEI VSG, Bordeaux, France) for segmentation. Fractures were isolated using a top-hat transform, and compression zones segmented using a grey scale-based isosurface.

#### 2.4.5. X-ray diffraction analysis

X-ray diffraction analysis was performed on sample 1, which was deemed representative as all samples originate from the same block in the same quarry, and this sample was not contaminated by the Wacker OH 100 consolidant or bullet residues such as lead because it was not shot. The analysis was carried out with a Philips PW1710 Automated Powder Diffractometer using Cu Kα radiation at 35 kV and 40 mA, between 2 and 70° 2*θ* at a scan speed of 0.04° 2*θ* s^−1^. Weighting factors were applied where necessary to calculate percentages of element present in the sample.

## 3. Results

### 3.1. X-ray diffraction analysis

The following mineral species were identified within sample 1: quartz (48%), gypsum (17%), calcite (13%), muscovite (13%), kaolinite (6%) and clinochlore (3%). The presence of clay, particularly muscovite and kaolinite, is of interest as the plastic deformation capacity of clay was shown to be of influence on the post-impact geometry of the mineral matrix (see microscopy results in §3.5), a mechanism previously reported by Fossen *et al*. [33] who related higher clay content within sandstone with higher deformation capacity.

### 3.2. Material loss

**Figure 5.**
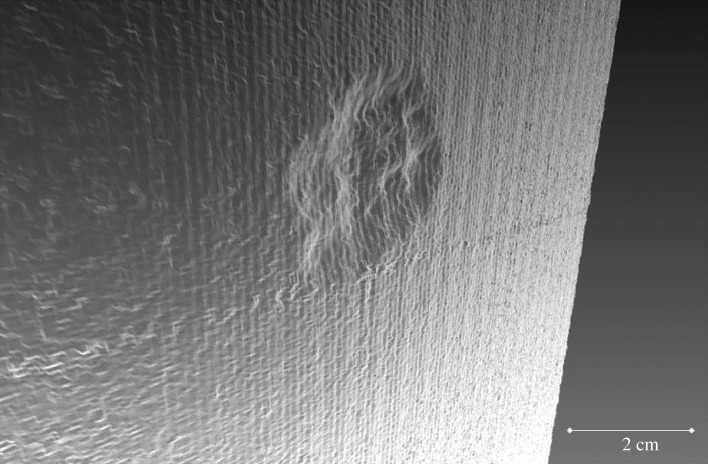
Mephisto Eos 3D scan of a bullet impact on the stone surface (not treated with Wacker OH 100).

Material loss from the surface after the bullet impact was almost negligible, as it averaged 2.57 g (0.05% of total weight) per sample (table 2), though loss of surface was clearly visible, as illustrated by figure 5. Interestingly, there was no noticeable difference reported between loss of mass from Wacker OH 100 treated surfaces and non-treated surfaces.

**Table 2.** Loss of weight of samples.

	pre-impact	post-impact	% change
1	3720.32	3720.32	0
2	3931.37	3928.9	0.062828
3	3770.47	3767.88	0.068692
4	4107.18	4104.87	0.056243
5	4136.8	4136.8	0
6	3976.4	3973.81	0.065134
7	4184.99	4182.69	0.054958
8	4091.14	4088	0.076751

### 3.3. Rock surface hardness

**Figure 6.**
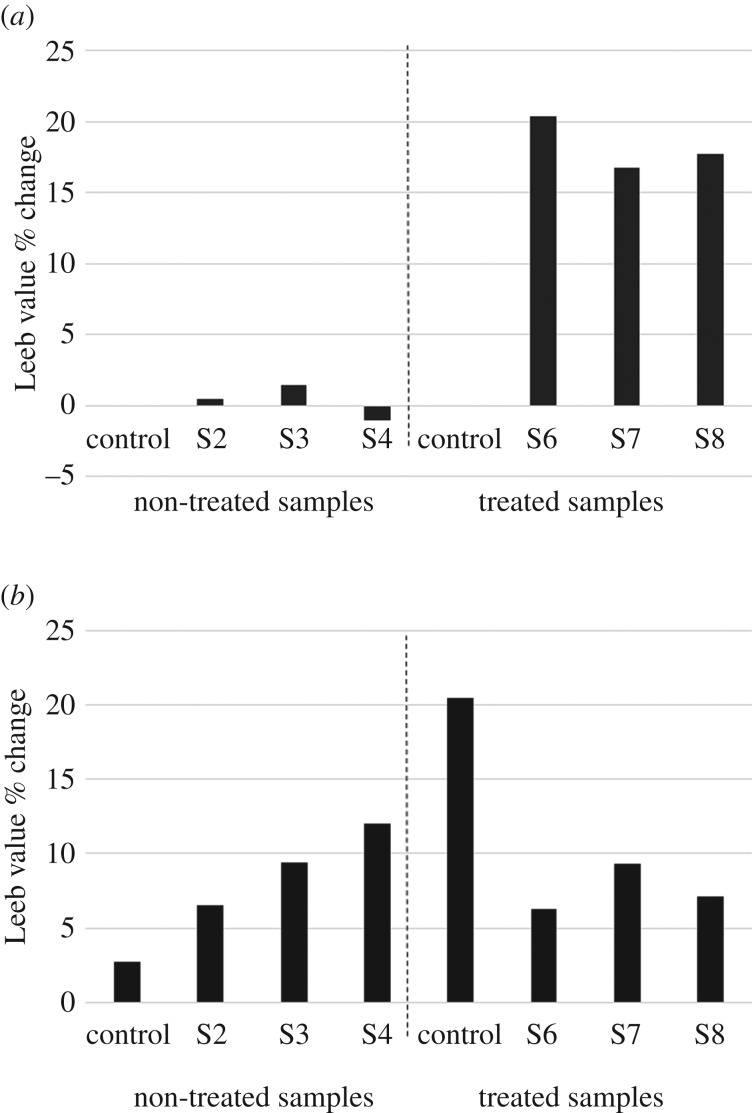
(*a*) Loss of strength of rock surface as measured after bullet impact and (*b*) loss of strength of rock surface as measured after environmental stress cycles in cabinet.

The repeated measurements of the sample surfaces are shown in figure 6. The initial Wacker OH 100 treatment shows a significant increase in the measured surface hardness at samples 5–8. This fits the original hypothesis that one treatment with Wacker OH 100 could be used to simulate the cementation of the surface associated with case hardening.

However, this increase in hardness appears to be rapidly reversed at the time of impact, while the overall surface hardness in samples 2–4 does not appear to reduce as dramatically, staying either roughly even (strength loss of 0.40% in sample 2 and 1.44% in sample 3) or in the case of sample 4 strangely increasing a fraction (1.04%). To interpret this data the elasticity of the rock face needs to be taken into account; while Wacker OH 100 can protect the surface from overall weathering processes such as abrasion and infiltration of moisture, owing to its hydrophobic nature [26], it can also be a deterioration factor as the loss of elasticity within the stone surface can severely decrease the overall stability of the surface upon impact. This phenomenon has been previously noted by Momber [34] who concluded that harder surfaces (as simulated by the Wacker OH 100 treatment) are prone to developing fracture networks upon impact whereas in softer materials (as simulated here by the untreated surfaces) crack formation was dampened by plastic flow of the impact resonance. It, therefore, appears that while the Wacker OH 100 treatment initially strengthened the surface, the noticeably lowered rock surface hardness values indicate that the impacts have created a network of microfractures across the rigid surface.

A further investigation of the overall results on strength loss (figure 6) indicates some unexpected results, while the impact of the initial bullet had a far greater impact on loss of strength on the surface- hardened samples, this trend reversed under the environmental strains placed upon the stone samples in the environmental cabinet, where environmental strain as recorded by surface strength loss had a greater impact on the non-treated samples. The presence of water at the foot of the block (samples 3, 4, 7 and 8) was detrimental irrespective of the presence of case hardening.

**Figure 7.**
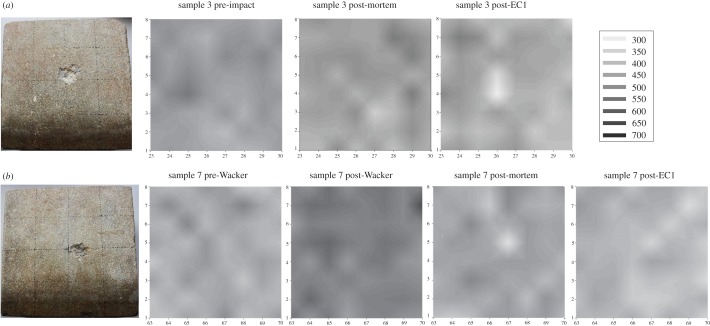
Piccolo data from samples 3 (*a*, sample not treated with Wacker OH 100) and 7 (*b*, sample treated with Wacker OH 100 and shot) in Leeb value. Values were interpolated using SigmaPlot area plot.

However, mean values cannot adequately describe spatial variations in elastic properties. Therefore, the Piccolo data from samples 3 and 7 measured across the surface of the block have been plotted in figure 7 to show change during the various stages of the experiment. Here, two interesting trends emerge. Firstly, sample 3, which was not treated with Wacker OH 100, does not show weakening directly corresponding to the impact area, although damage is apparent elsewhere. This follows the trend of the absorption of the impact by the more elastic nature of the surface (compared with the Wacker OH 100 treated surface). However, after the environmental cabinet treatment there is a clear weakening of the impact area, as represented by the lighter coloured area and relatively stable measurements around the impact area. This trend is reversed in sample 7 where initially the impact area is clearly identifiable post-impact (the very light grey area that corresponds with the location of the impact), accompanied by a general deterioration of the surface strength across the surface of the block. This impact area is much less visible after the environmental cabinet treatment. Instead there appears to be a general but less severe deterioration of the surface.

As can be seen in figures 6 and 7, all samples were negatively affected by the environmental cabinet treatment. However, the extent to which the samples were affected by the treatment appeared to be strongly related to the nature of fracture network developed at the time of impact, as based on the research set out by Momber [34]: The rigid, Wacker OH 100 treated, fractured surface appears to be more vulnerable to temperature changes overall, whereas the untreated and more plastic surface appears to cope far better with the thermal stresses placed upon the samples in the environmental cabinet but suffered noticeable degradation of the impact area, leaving it vulnerable to potential cavernous weathering. The pre-impact condition of the surface is therefore instrumental in determining the impact of a projectile, such as a bullet, and projecting future deterioration patterns.

### 3.4. Moisture movement

**Figure 8.**
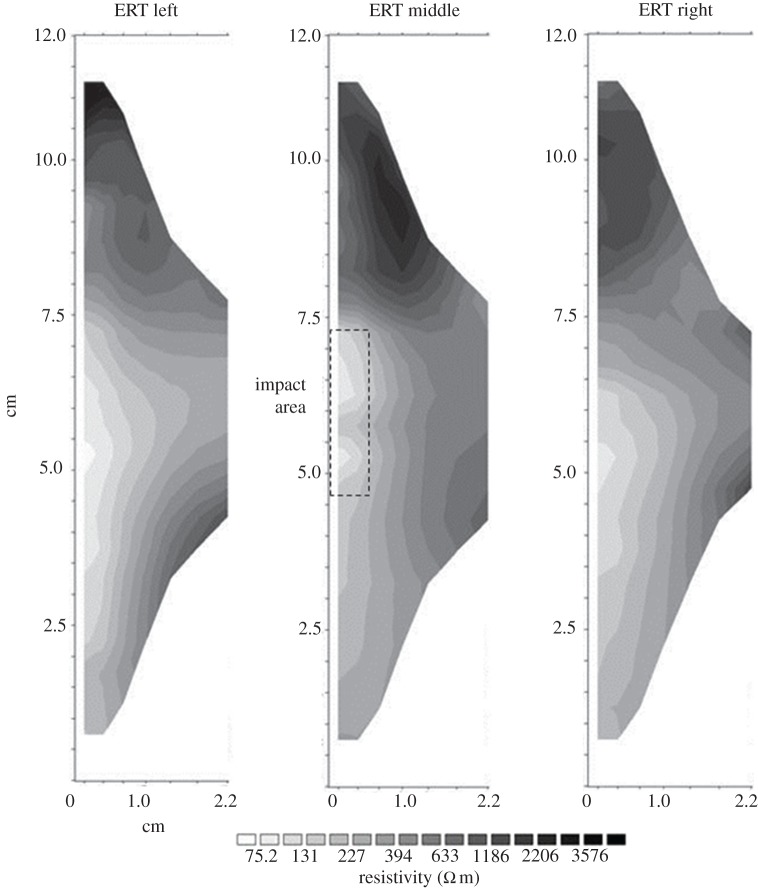
ERT transects of sample 3 (not treated with Wacker OH 100) after the environmental cabinet treatment.

Little is known about the relationship between internal moisture and impact areas resulting from armed warfare, even though internal moisture has been shown to be a driving weathering process [14]. The surface hardness experiment was therefore extended to include the behaviour of internal moisture under the conditions mapped out previously. Both samples 3 and 7 were immersed in water at the foot to simulate groundwater rise, with the impact site vertical and facing outwards as it would probably be situated within a damaged wall (figure 8), to a depth of 2 cm and are used here as examples to illustrate the processes found.

Resistivity measurements were taken along three transects across the stone sample (for sampling strategy, see figure 4). The middle transect covers the impact area and it was expected that the most noticeable changes would be seen in this area. Figure 8 shows the distribution of moisture in sample 3 after the environmental cabinet treatment. This sample was not treated with Wacker OH 100. The areas of low resistivity (light greys and whites) are assumed to indicate higher moisture content, whereas the dark grey and black areas indicate high resistivity, are assumed to be lower moisture contents. Capillary rise facilitates the upward movement of moisture until it reaches the impact area where it moves towards the surface and evaporates. An accumulation of moisture within the subsurface impact area can also be distinguished, forming a wedge shape of low resistivity into the stone body and saturating the inter-grain spaces in the fracture network. At the middle transect, the direct impact area shows a higher moisture content than in the surrounding area, suggesting that the evaporation is indeed concentrated in this area, as previously suggested.

**Figure 9.**
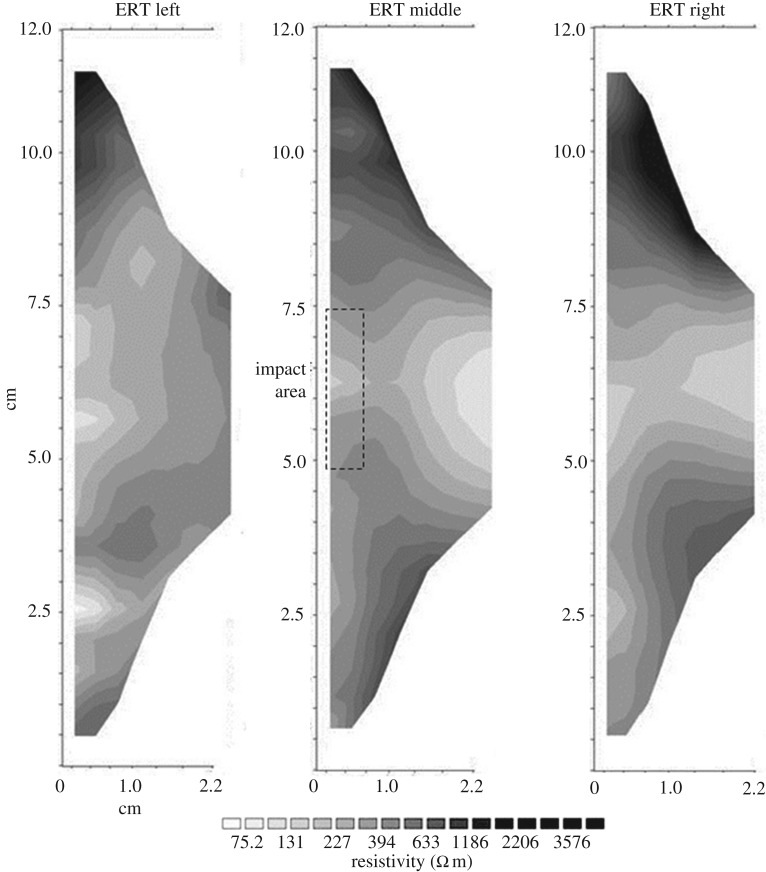
ERT transects of sample 7 (Wacker OH 100 treated) after the environmental cabinet treatment.

Sample 7 (figure 9) shows a very different pattern; moisture is drawn throughout the face of the stone, rather that drawn up towards the impact area. The impact area, while visible, is less clearly defined. However, the area behind the surface impact area clearly draws moisture in to a much greater degree than that seen in sample 3. Towards the foot of the block the moisture appears to be drawn up rather than into the block, as visible in sample 3, indicating more rapid capillary rise along the surface.

### 3.5. Microscope analysis

#### 3.5.1. Impact area development

**Figure 10.**
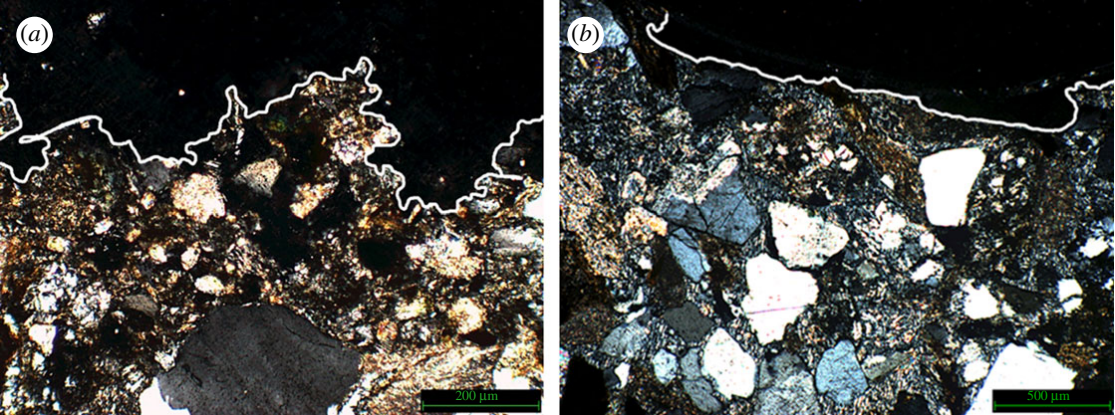
(*a*) Non-consolidated impact area (×4) and (*b*) consolidated impact area (×4) White line has been added to emphasize shape of impact area.

Before assessing the micro-scale repercussions of bullet impacts, we first investigated the overall creation of the crater, especially in relation to consolidated versus non-consolidated surfaces. We consistently observed that in the impacts created in non-consolidated stone the impact area was far more ‘jagged’, as illustrated by figure 10*a*, in contrast with the consolidated stone where the impact created a shallower and smooth curve (figure 10*b*).

#### 3.5.2. Quartz fragmentation

**Figure 11.**
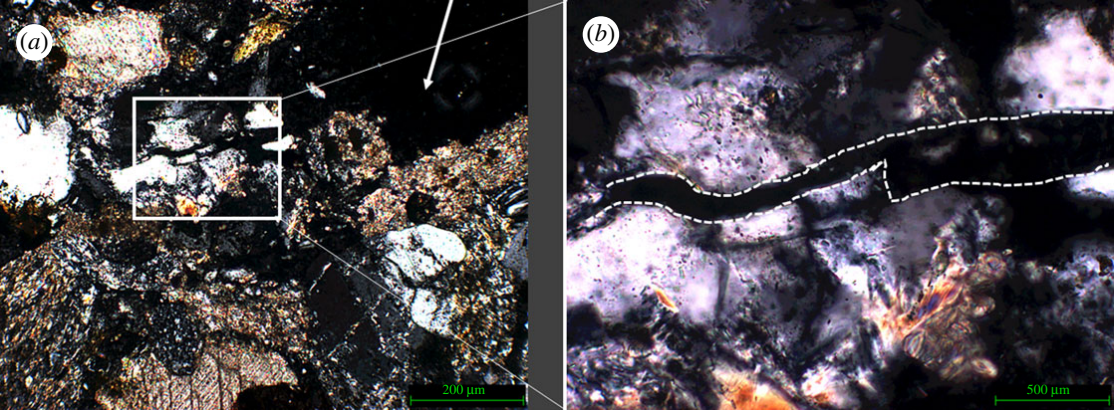
(*a*) Part of the impact area of sample 2 (not Wacker OH 100 treated); arrow indicates direction of impact. (*b*) Close-up of the fragmented and separated quartz found in the impact zone of sample 2, and white dashed lines clarify the position of the separation.

Quartz in the direct impact zone showed noticeable fracturing along the impact area. Figure 11*a* illustrates the fractures observed in the impact zone of sample 2 which was not treated with a consolidant. The absence of this surface support has resulted in the separation of the quartz fragment along the fracture line. Figure 11*b* shows a close-up of the area.

**Figure 12.**
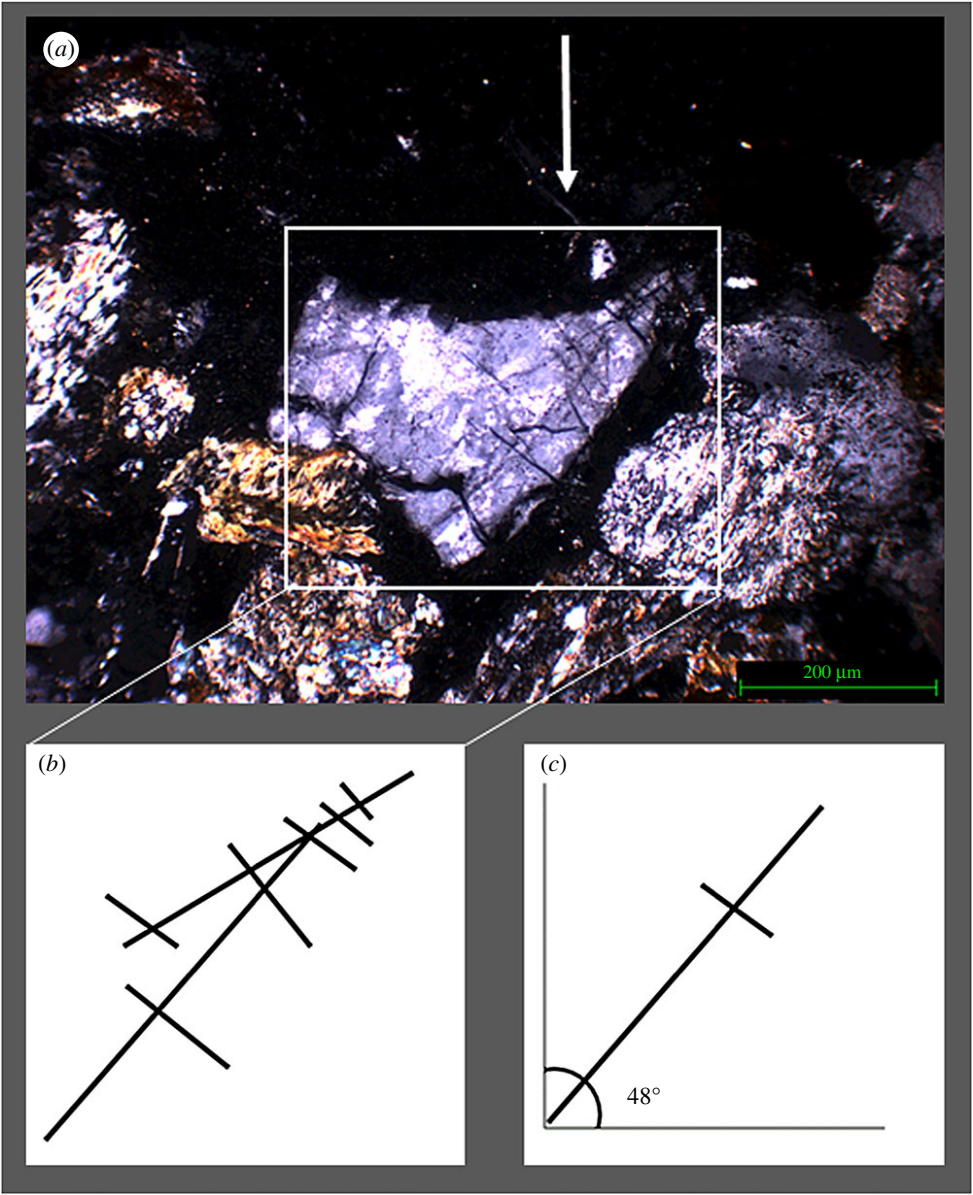
(*a*) Sample 3 (not treated with Wacker OH 100) impact area, white arrow indicates impact direction, (*b*) schematic of geometric fracture pattern and (*c*) schematic of angle of fracture network.

The bullet impacted in the area directly to the right of the fractured quartz, therefore, the conclusion could be drawn that the fracture and subsequent separation of the crystal is the result of the shockwaves generated at impact travelling sideways through the near-surface of the stone sample. The absence of a consolidant, and therefore a higher relative elasticity of the surface, facilitates sideways movement of the crystals. This type of separation was not observed in the samples treated with consolidant. This observation supports the notion that the nature of a micro-fracture network created upon impact is as dependent on the state of the material pre-impact as it is on the type of impact. As figure 12*a* illustrates, sample 3 exhibits this type of fracture network in the area directly adjacent to the impact. Figure 12*b*,*c* illustrates the geometric nature of the fracture network.

**Figure 13.**
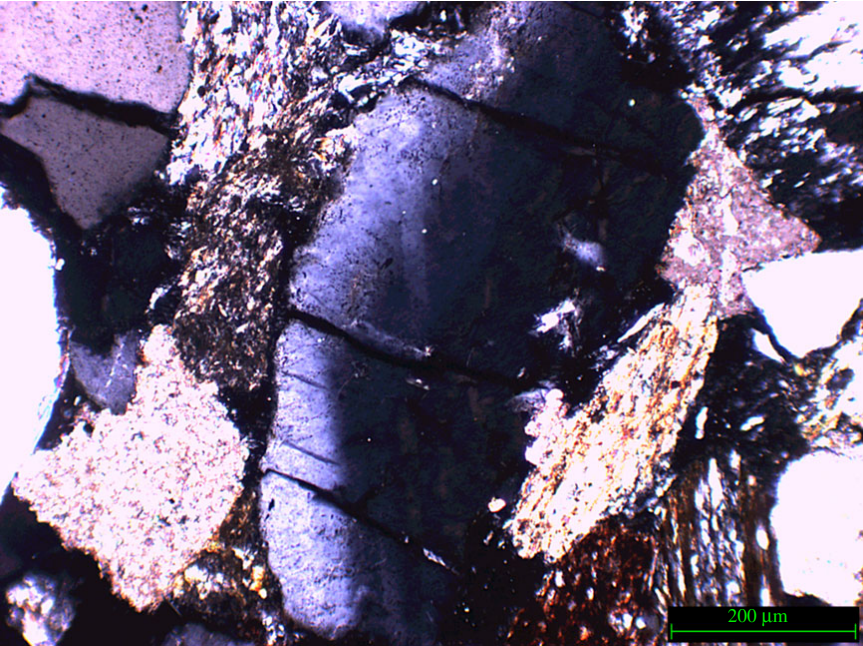
Sample 3, example of grain bruising.

Figure 13 illustrates further the fracture networks which were observed in sample 3 around the zone of impact. This sample also exhibits ‘bruising’ where part of the grain appears to have been reduced in refractive index (also noted by Fratanduono *et al.* [35] in shocked quartz), resulting in partial extinction as observed using cross-polarized light. This discoloration was not observed in grains outside of the direct impact area, indicating that this ‘bruising’ is closely connected to the shock generated on impact.

**Figure 14.**
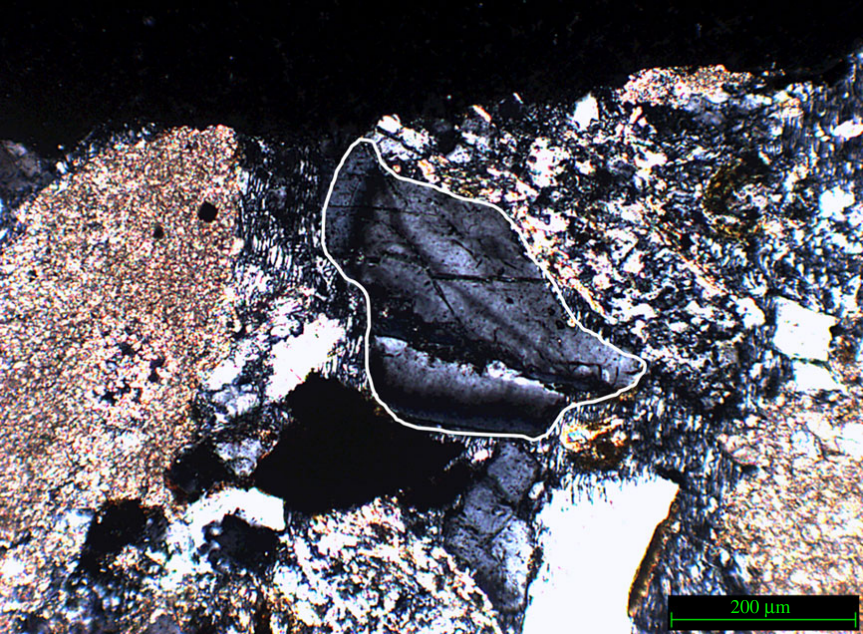
Sample 6 showing fractures, similar to those found in the non-consolidated samples. The mineral specifically referred to is outlined in white.

The above examples are observed in non-consolidated material. In consolidated material (samples 5–8) similar fractures were observed, implying that even though impact area development may differ the effects of the impact on mineral grains immediately surrounding the impact area are very similar. Figure 14 shows fractures found on the edge of the direct impact area in a Wacker OH 100 treated block (sample 6).

#### 3.5.3. Realignment of the clay matrix

**Figure 15.**
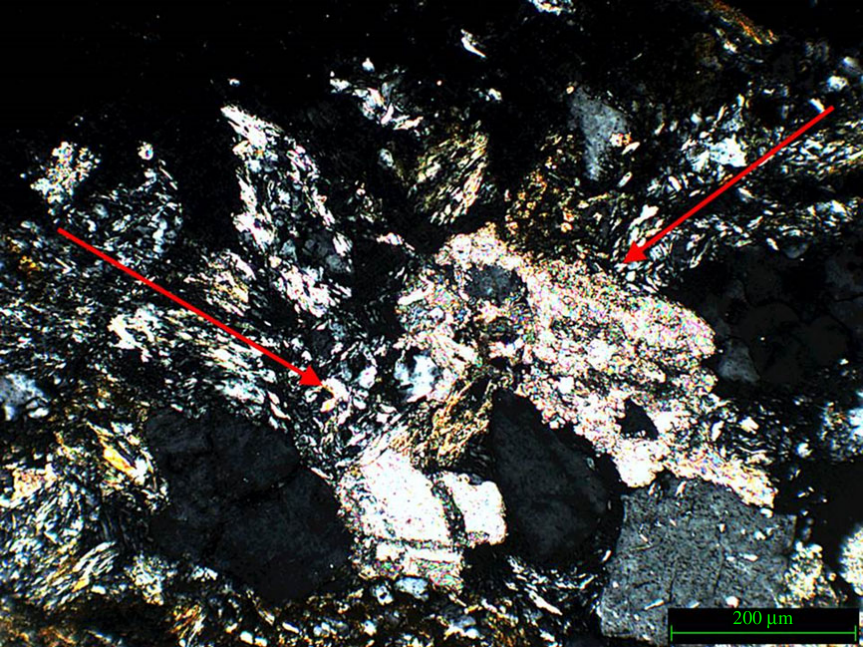
Sample 4, with arrows indicating clay matrix movement. This sample was not treated with Wacker OH 100.

Deformation of the clay (muscovite/kaolinite) cementation indicates a realignment of the general matrix in response to the shock generated on impact. Figure 15 illustrates this realignment in relation to the centre of the bullet impact which is in the area between the two arrows. The arrows indicate the new direction of the clay minerals, an alignment which is not visible in the non-impacted samples

#### 3.5.4. Three-dimensional X-ray tomographic analysis of impact planes

**Figure 16.**
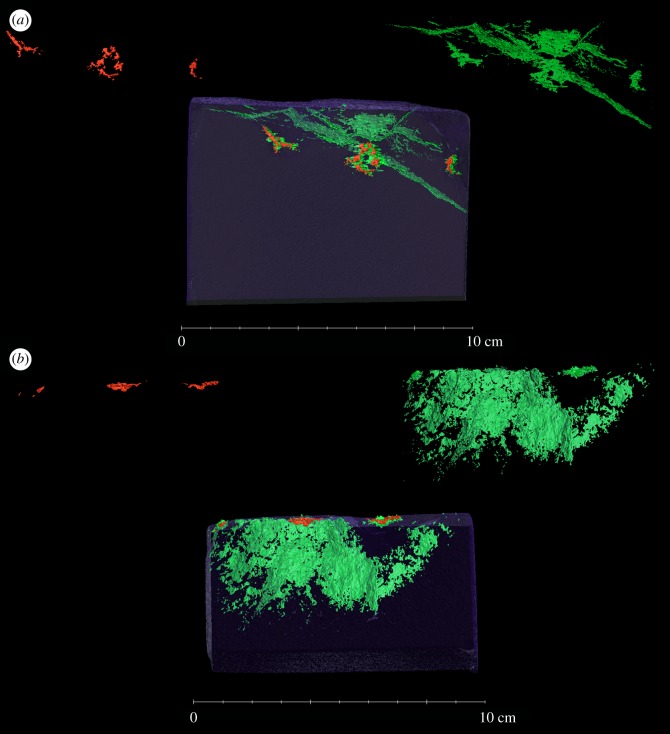
X-ray of impact areas, green demarcates areas of low density (fractured), whereas red indicates areas of higher density, likely to be lead deposits from the bullet encasing. (*a*) Frontal view (line of sight parallel to travel of bullet) and (*b*) top-down view. This sample was not treated with Wacker OH 100.

As all previous tests indicated both deformation of minerals around the impact zone as well as consolidation of the clay, a sample was scanned using X-ray tomography to map out compaction and fracture zones. These results are shown in figure 16, where the impacts coincide with compacted areas, whereas the fractured area extends onto the surface, radiating out from underneath the impact zones. The damage appears to have been caused by stress travelling into the block as the bullet hit, towards the edges of the block while following a bedding plane within the stone sample. This exploitation of a pre-existing weakness is a commonly observed mechanism in stone under environmental stress [36]. These results also confirm the creation of compaction areas at the site of impact, which in turn affects impact site response to environmental stress and potential for long-term deterioration.

## 4. Discussion

As shown in §3.2 the mass of material loss at the time of impact was nominal as would be expected from a 0.22 calibre impact. However, surface hardness and ERT measurements indicate that the impact of a small projectile, such as a 0.22 calibre bullet, can greatly alter the behaviour of a stone under changing environmental circumstances. While the Wacker OH 100 was successful in raising the surface hardness, simulating the surface cementation created by case hardening, the increase in strength was negated after the projectile impact as surface strength was substantially lowered again. The non-treated samples did not show such surface-wide deterioration but instead showed a noticeable lowering of surface hardness at the bullet impact site. This resulted in very different moisture regimes as the treated sample appeared to conduct capillary rise of moisture along the surface, whereas the non-treated sample showed further ingress of moisture into the body of the stone. Considering the noticeable decrease in surface hardness after the impact, it is likely that the fracture network at and near the surface facilitates this capillary rise. While this is based on a simulation of case hardening, rather than naturally hardened surfaces, there are nonetheless lessons that can be learnt. Wacker OH 100 can increase flexural strength within a stone mass [37], which is useful when the stone is under physical pressure, but decreases its ability to transmit shockwaves with minimal damage. Given this lack of plasticity near the surface, it is possible that the area directly behind the impact can severely weaken. Assuming that moisture follows the available pathways, i.e. the fracture networks, a continued deterioration of the surface over time owing to increased evaporation and capillary rise of groundwater could potentially be a contributing factor to future acceleration of surface deterioration.

There are potential similarities between shocked features from meteorite impacts and the impact of bullets. Previous literature has investigated the phenomenon ‘shocked quartz’ which is commonly found in sites that have been affected by meteorite strikes [38–40]. The heat and shockwaves generated by such impacts create a tell-tale network of parallel and perpendicular microfractures, and deformation of the mineral through (partial) melting. Even at this low calibre impact investigated here these changes were observed within our samples.

Quartz grains that have been shocked in meteorite impacts exhibit a variety of characteristic deformation features. Planar deformation features (PDFs) and planar fractures (PFs) are the most widely recognized. The former are planes of amorphous SiO_2_ lamellae nanometres in width with spacings of 2–10 µm that occur in specific crystallographic orientations [41]; or Brazil twins [42], whereas PFs are open or closed fractures with wider spacings than PDFs [43]. Less well characterized are a variety of non-planar and unorientated fractures that are common in shocked minerals and ubiquitous in shocked quartz [42]. Other shock effects in quartz include mosaicism, amorphization and polymorphic transformations.

**Figure 17.**
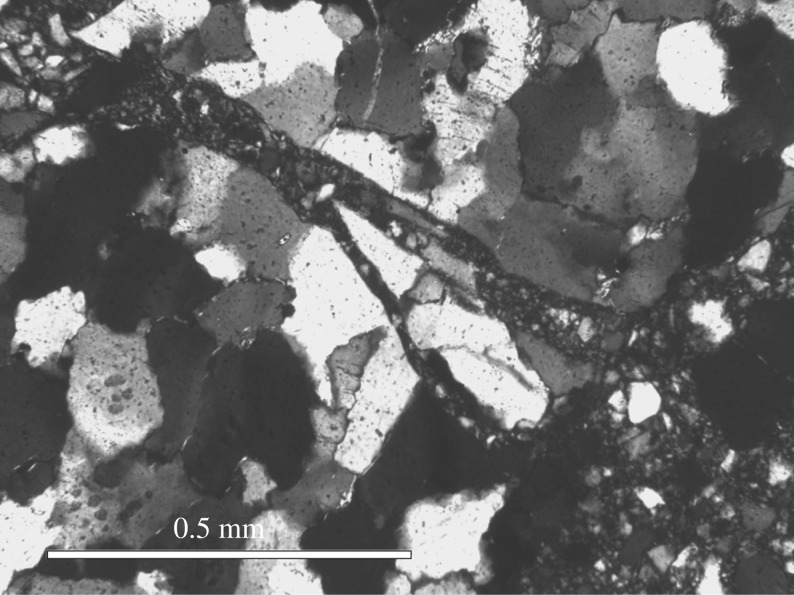
Fractures in a quartz-rich metasedimentary rock from the Ries Impact structure, Germany. Compare fractures with figures 10–14. Crossed polars. Sample from Alte Bürg quarry, 12 km from impact centre.

The fractures observed in quartz under the bullet impacts are too coarse to be classified as PDFs or PFs, but they fit well with the description of fractures caused by rarefraction waves at pressures less than the elastic limit of quartz, below 5 GPa [42,43]. Figure 17 shows an example of typical fractures found in quartz metasedimentary rocks from the Ries Impact structure, Germany, which have similarities to the fractures seen in figures 10–14. The extinction patterns observed under cross-polarized light in quartz grains are potentially similar to mosaicism [44], although more detailed characterization is necessary to establish this similarity. In any case, the extinction patterns can be clearly related to the shot by their localization near the bullet impact site. These observations suggest that the deformation caused by bullets has some similarities with meteorite shock damage, and could be considered to occur at the low-pressure end of the spectrum of shock-induced microstructures. This comparison could have useful implications for further studies of arms-induced damage.

The microscopy presented here shows that even relatively small 0.22 calibre bullet impacts alter the substrate significantly, both through removal of material and alteration of the grains within the direct impact area. The deformation is noticeable and appears to lead to both crack formation and compaction within the impact area. The presence of kaolinite facilitates the compaction of the impact area, thereby potentially hampering internal moisture flows. The relatively high percentage of clay minerals (muscovite 13% and kaolinite 6%) facilitates this compressive cementation, leading to reduced movement of moisture through this section of the stone surface. The long-term effect could be the development of weak areas around the impact zone where crack formation facilitates more rapid through-flow of moisture while clay deformation further concentrates through-flow, thereby causing larger areas of deterioration than previously estimated. However, the clean removal of surface material in Wacker OH 100 treated samples, in contrast with the more ‘jagged’ and fragile removal of material in non-consolidated samples implies that the consolidation provides some measure of protection for the subsurface; the impact is intercepted by the consolidated surface, causing a fracture between the consolidated area and the subsurface followed by a removal of any consolidated material. In non-consolidated material, no such boundary exists and instead the fracture paths may depend on pre-existing weaknesses in the material such as around grains in particularly porous areas. More research will be needed in this area before firm conclusions can be drawn.

The above results have a number of potential implications for stone conservation. Somewhat counterintuitively, any monuments which have been treated with consolidation substances or have developed a surface hardened by natural cementation during years of exposure could be at greater risk of deterioration after a projectile impact owing to fracturing of the surface as a result of decreased plasticity. While the damage on the surface may only be visible at the direct impact point, the weakening of the stone may be far more widespread through the material than previously realized. In addition, subsequent alteration in internal moisture behaviour could result in significant problems for the conservation of heritage as previously dominant weathering processes could be altered to suit the new moisture regime. While formation of fracture networks owing to projectile impact is not a new concept, this research places it in a new context by combining experimental weathering and impact studies that tend to be restricted to engineering investigations, and setting it in the context of the complexity of heritage in conflict areas. Armed warfare is becoming an increasing threat to heritage as the availability of weapons and their impact potential increase. These tests are based on relatively small 0.22 calibre bullet impacts with minimal surface material loss, yet the effects were far greater than appreciated from a visual inspection. The results from this study, therefore, beg the question: if such small impacts can alter the stone to this extent, what are the long-term consequences of larger impacts such as AK-47s?

## 5. Concluding remarks

While this report gives a preliminary indication of the influence of projectile impacts on the response of stone to temperature and moisture ingress, further research is needed to comprehend this rather complex issue. Additional factors, such as salt weathering, loss of tensile strength throughout the stone mass and the effects of larger, higher-velocity impacts need to be investigated further before drawing firm conclusions about the influence of projectile impacts on conservation strategies in armed warfare zones. Further research into the response of other common building materials such as brick and concrete is necessary to map damage in built heritage sites made of composite materials. The complexity of the response of fracture networks to environmental strain, even at a small calibre scale, is evident from this work. This study also illustrates the need for a multidisciplinary approach to the long-term impacts of armed conflict.

## Supplementary Material

AK47 impact interactive file

## Data accessibility

All data are available either directly from the lead author (lisa.mol@uwe.ac.uk) or can be downloaded from the Dryad Digital Repository: http://dx.doi.org/10.5061/dryad.f5d20 [45].

## References

[1] Ghaidan U 2008 Damage to Iraq's wider heritage. In The destruction of cultural heritage in Iraq (eds Stone PG, Bajjaly JF). Woodbridge, UK: Boydell Press.

[2] Harmanşah Ö 2015 ISIS, heritage, and the spectacles of the destruction in the global media. Near East. Archaeol. 78, 170–177. (doi:10.5615/neareastarch.78.3.0170)

[3] British Academy 2010 Fighting a looting battle? See http://www.britac.ac.uk/news/news.cfm/newsid/16.

[4] Project Mosul 2016 See http://projectmosul.org/.

[5] Guillemette A 2013 Coming together at Easter: commemorating the 1916 Rising in Ireland, 1916–1966. Unpublished PhD thesis, Concordia University, Canada.

[6] Hamber B 2004 *Public memorials and reconciliation processes in Northern Ireland*. In Paper presented at the Trauma and Transitional Justice in Divided Societies Conf, Airlie House, Warrington, VA, USA, 27–29 March 2004.

[7] Mahfuz H, Zhu Y, Haque A, Abutalib A, Vaidya U, Jeelani S, Gama B, Gillespie J, Fink B 2000 Investigation of high-velocity impact on integral armor using finite element method. Int. J. Impact Eng. 24, 203–217. (doi:10.1016/S0734-743X(99)00047-0)

[8] Barauskas R, Abraitienė A 2007 Computational analysis of impact of a bullet against the multilayer fabrics in LS-DYNA. Int. J. Impact Eng. 34, 1286–1305. (doi:10.1016/j.ijimpeng.2006.06.002)

[9] Børvik T, Olovsson L, Dey S, Langseth M 2011 Normal and oblique impact of small arms bullets on AA6082-T4 aluminium protective plates. Int. J. Impact Eng. 38, 577–589. (doi:10.1016/j.ijimpeng.2011.02.001)

[10] Børvik T, Dey S, Olovsson L 2015 Penetration of granular materials by small-arms bullets. Int. J. Impact Eng. 75, 123–139. (doi:10.1016/j.ijimpeng.2014.07.016)

[11] Wang MY, Shanbiao Z, Daliang Z 2014 Calculation of depth of projectile penetration into rock. In Projectile impact: modelling techniques and assessment of target material (ed. Syngellakis S), pp. 183–190. Southampton, UK: WIT Press.

[12] CCI (no date) The ten agents of deterioration: physical forces. See http://canada.pch.gc.ca/eng/1444924113472 (accessed 14 November 2016).

[13] Viles HA, Goudie A 2004 Biofilms and case hardening on sandstone from Al-Quwayra, Jordan. Earth Surf. Process. Landf. 29, 1473–1485. (doi:10.1002/esp.1134)

[14] Mol L, Viles HA 2013 Exposing drying patterns: using electrical resistivity tomography to monitor capillary rise in sandstone under varying drying conditions. Environ. Earth Sci. 68, 1647–1659. (doi:10.1007/s12665-012-1858-x)

[15] McAlister JJ, Smith BJ, Curran JA 2003 The use of sequential extraction to examine iron and trace metal mobilisation and the case-hardening of building sandstone: a preliminary investigation. Microchem. J. 74, 5–18. (doi:10.1016/S0026-265X(02)00043-7)

[16] Hack HRGK, Hingira J, Verwaal W 1993 Determination of discontinuity wall strength by Equotip and ball rebound tests. Int. J. Rock Mech. Min. Sci. Geomech. Abs. 30, 151–155. (doi:10.1016/0148-9062(93)90707-K)

[17] Verwaal W, Mulder A 1993 Estimating rock strength with the Equotip hardness tester. Int. J. Rock Mech. Min. Sci. Geomech. Abs. 30, 659–662. (doi:10.1016/0148-9062(93)91226-9)

[18] Viles H, Goudie A, Grab S, Lalley J 2011 The use of the Schmidt Hammer and Equotip for rock hardness assessment in geomorphology and heritage science: a comparative analysis. Earth Surf. Process. Landf. 36, 320–333. (doi:10.1002/esp.2040)

[19] Dorn RI et al. 2012 Case hardening vignettes from the western USA: convergence of form as a result of divergent hardening processes. Yearb. Assoc. Pac. Coast Geogr. 74, 53–75. (doi:10.1353/pcg.2012.0003)

[20] Mol L, Viles HA 2012 The role of rock surface hardness and internal moisture in tafoni development in sandstone. Earth Surf. Process. Landf. 37, 301–314. (doi:10.1002/esp.2252)

[21] Conca JL, Rossman GR 1982 Case hardening of sandstone. Geology 10, 520–523. (doi:10.1130/0091-7613(1982)10<520:CHOS>2.0.CO;2)

[22] McKinley JM, Warke PA 2007 Controls on permeability: implications for stone weathering. Geol. Soc. Lond. Spec. Publ. 271, 225–236. (doi:10.1144/GSL.SP.2007.271.01.22)

[23] Alexandrowicz Z, Marszałek M, Rzepa G 2014 Distribution of secondary minerals in crusts developed on sandstone exposures. Earth Surf. Process. Landf. 39, 320–335. (doi:10.1002/esp.3449)

[24] Mosquera MJ, Pozo J, Esquivias L 2003 Stress during drying of two stone consolidants applied in monumental conservation. J. Sol-Gel Sci. Technol. 12, 1227–1231. (doi:10.1023/A:1020776622689)

[25] Moropoulou A, Haralampopoulos G, Tsiourva Th, Auger F, Birginie M 2003 Artificial weathering and non-destructive tests for the performance evaluation of consolidation materials applied on porous stones. Mater. Struct. 36, 201–217. (doi:10.1007/BF02479613)

[26] Da Costa DMR, Rodrigues JD 2011 The effect of water on the durability of granitic materials consolidated with ethyl silicates. In *Proc. CCI Symp. ICC 2011—adhesives and consolidants for conservation: research and applications, Ottawa, Canada, 17–21 October 2011*.

[27] Kemp J 2006 Marble. In Stone conservation: principles and practice (ed. Henry A), pp. 217–236. Abingdon, UK: Routledge.

[28] Myrin M, Malaga K 2006 A case study on the evaluation of consolidation treatments of Gotland sandstone by use of ultrasound pulse velocity measurements. In Heritage, weathering and conservation volume II (eds Fort R, Alvarez de Buergo M, Gomez-Heras M, Vazquez-Calvo C), pp. 749–755. London, UK: Taylor & Francis.

[29] Mol L, Viles HA 2010 Geoelectric investigations into sandstone moisture regimes: implications for rock weathering and the deterioration of San Rock Art in the Golden Gate Reserve, South Africa. Geomorphology 118, 280–287. (doi:10.1016/j.geomorph.2010.01.008)

[30] Shockey DA, Curran DR, De Carli PS 1975 Damage in steel plates from hypervelocity impact. I. Physical changes and effects of projectile material. J. Appl. Phys. 46, 3766–3775. (doi:10.1063/1.322162)

[31] Sass O, Viles HA 2006 How wet are these walls? Testing a novel technique for measuring moisture in ruined walls. J. Cult. Herit. 7, 257–263. (doi:10.1016/j.culher.2006.08.001)

[32] Mol L, Preston PR 2010 The writing's in the wall: a review of new preliminary applications of electrical resistivity tomography within archaeology. Archaeometry 52, 1079–1095. (doi:10.1111/j.1475-4754.2010.00516.x)

[33] Fossen H, Schultz RA, Shipton ZK, Mair K 2007 Deformation bands in sandstone: a review. J. Geol. Soc. Lond. 164, 755–769. (doi:10.1144/0016-76492006-036)

[34] Momber AW 2004 Deformation and fracture of rocks due to high-speed liquid impingement. Int. J. Fract. 130, 683–704. (doi:10.1007/s10704-004-2507-5)

[35] Fratanduono DE, Eggert JH, Boehly TR, Barrios MA, Meyerhofer DD, Jensen BJ, Collins GW 2011 Index of refraction of shock-released materials. J. Appl. Phys. 110, 083509 (doi:10.1063/1.3650258)

[36] Twidale CR, Bourne JA 2007 Fractures as planes of dislocation and two-way translocation: their significance in landform development. Phys. Geogr. 28, 193–217. (doi:10.2747/0272-3646.28.3.193)

[37] Pinto APF, Rodrigues JD 2008 Stone consolidation: the role of treatment procedures. J. Cult. Herit. 9, 38–53. (doi:10.1016/j.culher.2007.06.004)

[38] Miura Y 1991 Evidence for shock wave effect of meteoritic impact. Shock Waves 1, 35–41. (doi:10.1007/BF01414866)

[39] Riog CI, Cavosie AJ, McDougal DJ, Cordua WS, Mattson C 2013 Detrital shocked quartz in modern sediments eroded from the Rock Elm impact structure, Wisconsin. In *44th Annu. Lunar and Planetary Science Conf.*, vol. 2685. Houston, TX: Lunar and Planetary Institute.

[40] Thomson OA, Cavosie AJ, Moser DE, Barker I, Radovan HA, French BM 2014 Preservation of detrital shocked minerals derived from the 1.85 Ga Sudbury impact structure in modern alluvium and Holocene glacial deposits. Geol. Soc. Am. Bull. 126, 720–737. (doi:10.1130/B30958.1)

[41] Alexopoulos JS, Grieve RAF, Robertson PB 1988 Microscopic lamellar deformation features in quartz: discriminative characteristics of shock-generated varieties. Geology 16, 796–799. (doi:10.1130/0091-7613(1988)016<0796:MLDFIQ>2.3.CO;2)

[42] Stöffler D, Langenhorst F 1994 Shock metamorphism of quartz in nature and experiment: I. Basic observation and theory. Meteoritics 29, 155–181. (doi:10.1111/j.1945-5100.1994.tb00670.x)

[43] French BM 1998 Traces of catastrophe: a handbook of shock-metamorphic effects in terrestrial meteorite impact structures. LPI contribution no. 954 Houston, TX: Lunar and Planetary Insitute, 120 pp.

[44] Ernstson K, Claudin F n.d. Shock metamorphism page. See http://www.impact-structures.com/shock-metamorphism-page/ (accessed 9 May 2016).

[45] Mol L, Gomez-Heras M, Brassey C, Green O, Blenkinsop T 2017 Data from: The benefit of a tough skin: bullet holes, weathering and the preservation of heritage. Dryad Digital Repository. (doi:10.5061/dryad.f5d20) 10.1098/rsos.160335 PMC5367304 28386411

