# Supplementary material for: The benefit of a tough skin: bullet holes, weathering and the preservation of heritage
Source: R Soc Open Sci. 2017 Feb 22;4(2):160335. doi: 10.1098/rsos.160335 (PMC5367304; doi:10.1098/rsos.160335)
Supplement: AK47 impact interactive file [file rsos160335supp1.pdf]

Generated with Agisoft PhotoScan
